# *LcNAC13* Is Involved in the Reactive Oxygen Species-Dependent Senescence of the Rudimentary Leaves in *Litchi chinensis*

**DOI:** 10.3389/fpls.2022.886131

**Published:** 2022-05-09

**Authors:** Congcong Wang, Hao Liu, Lijie Huang, Houbin Chen, Xingyu Lu, Biyan Zhou

**Affiliations:** ^1^Guangdong Litchi Engineering Research Center, College of Horticulture, South China Agricultural University, Guangzhou, China; ^2^Guangdong Provincial Key Laboratory of Silviculture, Protection and Utilization, Guangdong Academy of Forestry, Guangzhou, China

**Keywords:** *NAC*, rudimentary leaf, senescence, transcriptome, VIGS, litchi

## Abstract

Litchi is an important evergreen fruit tree. Floral formation in litchi is induced by low temperatures (LTs). However, unstable flowering is a challenge for litchi production in times of global warming and climate change. Previous studies have shown that the methyl viologen dichloride hydrate-generated reactive oxygen species (ROS) could promote flowering. Leaves in the panicles may affect the development of the inflorescence in litchi under high-temperature condition. In this study, potted litchi trees were transferred to growth chambers at LT and high temperature (HT). From a previous dataset of the RNA sequencing of the ROS-treated rudimentary leaves, a NAC transcription factor-encoding gene *LcNAC13* was identified. By genetic transformation of *LcNAC13* to *Arabidopsis thaliana* and tobacco, it was found that the ROS-induced senescence of the leaves was accelerated. Silencing *LcNAC13* by virus-induced gene silencing (VIGS) delayed ROS-dependent senescence. Our results suggested that *LcNAC13* regulates rudimentary leaf senescence. Our study provided a new target gene for the future molecular breeding of new cultivars that could flower under global warming conditions.

## Introduction

Litchi (*Litchi chinensis*) is an important evergreen fruit tree in southern Asia. Litchi floral buds are a mix of panicle primordia and rudimentary leaves (Yang et al., 2017). In winter and early spring, if temperatures are low enough, the rudimentary leaves (panicle leaves) abscise and the floral buds develop into pure panicles. However, in the near 20 years, warm winters due to global warming frequently happened. Under these conditions, the mixed buds might be exposed to relatively high temperatures (HTs), then the rudimentary leaves developed into fully expanded leaves, and the panicle primordia stopped developing and might shrink (Zhou et al., 2008). However, if the panicle leaves are killed or their growth is inhibited, the panicles still can develop. Actually, growers always use ethephon, an ethylene producer to control panicle leaf in litchi. Moreover, methyl viologen dichloride hydrate (MV), producer of the reactive oxygen species (ROS), and sodium nitroprusside, donor of nitric oxide (NO), are proved to be effective chemicals that can inhibit leaf growth (Zhou et al., 2012). Therefore, understanding the regulatory mechanism of panicle leaf senescence and abscission is important for flowering control in litchi.

We have previously studied the features of the low-temperature-(LT), ROS-, and NO-induced senescence of panicle leaves and found that programmed cell death (PCD) is involved in these processes (Zhou et al., 2008; Yang et al., 2018). We also constructed a litchi reference transcriptome for the ROS-treated young leaves using Illumina RNA sequencing (RNA-Seq) to identify ROS-responsive genes. We have identified a litchi homolog *MCII* (*LcMCII-1*) and showed that silencing *LcMCII-1* by virus-induced gene silencing (VIGS) delayed ROS-dependent senescence. Ectopic overexpression of *LcMCII-1* in Arabidopsis promoted ROS-dependent senescence of leaves as well as natural senescence (Wang et al., 2017).

NAC transcription factors (TFs) are plant-specific TFs (Zhang et al., 2018; Li et al., 2020). More than 100 members were found in *Arabidopsis thaliana* (Penfold and Buchanan-Wollaston, 2014). NAC was named after no apical meristem petunia (NAM), *A. thaliana* ATAF1/2, and CUC2 (Trupkin et al., 2019; Munir et al., 2020). NAC proteins widely regulate plant growth and development (Olsen et al., 2005; Yuan et al., 2019). Interestingly, more than 30 *NAC* genes showed enhanced expression during natural leaf senescence in *Arabidopsis* (Breeze et al., 2011).

Leaf senescence is the final stage of leaf development (Lin and Wu, 2004; Kim et al., 2018). The process of senescence may help the plant to aid in the adaptation to adverse environmental conditions (Schippers et al., 2015). Leaves may be artificially senescent before maturity when they are exposed to LTs and drought (Allu et al., 2014; Naschitz et al., 2014; Muñoz and Munné-Bosch, 2018). The panicle leaf senescence induced by ROS or LT is regarded as premature leave senescence (Yang et al., 2017, 2018). Up till now, little is known about the role of NACs in litchi panicle leaf senescence.

In this study, we used our previous RNA-Seq dataset of the ROS-treated rudimentary leaves (Lu et al., 2014) to identify candidate *LcNACs* potentially involved in panicle leaf senescence. We transferred the litchi potted trees to growth chambers for LT- and HT- treatments to induce senescing leaves and growing leaves, respectively. The expression profiles of the *NACs* in the senescing and developing panicle leaves were compared for further screening. Then, the identified *LcNAC13* was subjected to VIGS and transferred to Arabidopsis and tobacco (*Nicotiana benthamiana*) for functional analysis. Our work provided target genes for the future molecular breeding of new cultivars that could flower under global warming conditions.

## Materials and Methods

### Plant Materials and Experimental Procedures

Four-year-old air-layered ‘Nuomici’ litchi trees were grown in 30-L pots containing coconut chaff, loam, and mushroom cinder (v/v/v, 3:1:1). The trees were subjected to LT conditions for floral induction in open fields in winter. Once panicle primordia emerged, six replicate trees were transferred to a growth chamber at 12-h photoperiod and at 18°C (LT) to encourage panicle development and promote rudimentary leaf senescence. Another six trees were transferred to a growth chamber at 12-h photoperiod and at 26°C (HT) to encourage the growth of the rudimentary leaves. The percentage of the flowering terminal shoots, the axillary panicles per panicle, and the leafy panicles, the number of leaflets per panicle, and the number of fruits per panicle were determined.

For gene expression assay, panicle leaf development was divided into five stages. Senescing leaves and developing leaves at different developmental stages as described by Yang et al. (2017) were collected. Leaves were frozen in liquid nitrogen and stored at −80°C for total RNA extraction and gene expression determination.

For ROS treatment, potted trees were placed in a greenhouse at 26°C with a diurnal 12-h photoperiod. For spraying, 120 μM MV solution (Sigma) was used. After 0, 1, 3, and 5 days of MV treatment, rudimentary leaf samples were collected and stored at −80°C for total RNA extraction.

For the VIGS treatment, potted ‘Nuomici’ trees were transferred to a growth chamber at 12-h photoperiod, at 23°C, and with a relative humidity of 50 to 60%.

Colombian wild-type (Col-0) *A. thaliana* and *N. benthamiana* were chosen for the heterologous genetic transformation. Transgenic plants were grown under a photoperiod of 18 h, a day/night temperature of 22°C, and a photosynthetic photon density of 60 μmol/m^2^ s^1^. Rosette leaves of the 30-day-old transgenic Arabidopsis were cut and placed in Petri dishes, sprayed with 40 μM MV. SPAD values corresponding to the amount of chlorophyll in the leaves (Ling et al., 2011; Galanti et al., 2019) were measured with a chlorophyll meter (SPAD, model 502, Minolta, Japan) in 0, 3, and 6 days of treatment. To measure transgenic tobacco in response to ROS, leaves at 4 to 5 whorl were sprayed with 40 μM MV. Ten days after spraying, SPAD values of the fourth or fifth leaves were recorded.

### Identification of Reactive Oxygen Species-Responsive *NACs* Potentially Involved in Rudimentary Leaf Senescence

All the ROS-responsive *NACs* were screened from the RNA-Seq dataset of the ROS-treated rudimentary (Lu et al., 2014). The dataset is available from the NCBI Short Read Archive (SRA)^1^ under the number SAR158542.

### Quantitative RT-PCR Analysis

First-strand cDNA was synthesized by Reverse Transcriptase M-MLV (RNase H-) system (Takara, Japan) from 1 μg RNA. Primers for quantitative RT-PCR (qRT-PCR) were designed by Primer 6.0 software and synthesized by Sangon Co. Ltd. (Shanghai). The litchi homolog β*-actin* was selected as a reference gene (LcActin-F/R, Supplementary Table 1). qRT-PCR was performed on a CFX real-time PCR machine (Bio-Rad, United States) according to the method described by Lu et al. (2014).

### Cloning and Bioinformatics Analysis of *LcNAC13*

The *LcNAC13*-specific primers (LcNAC13-F/R, Supplementary Table 1) were designed based on our transcriptome data of the ROS-treated rudimentary leaves. Cloning of the target fragment was performed using the T/A cloned pMD18-T vector (Takara, Japan). The target gene sequence was analyzed using BLAST online program for comparative analysis^2^. CDD was used for the prediction of conserved structural domains of the target gene^3^. Multiple sequence alignment of NACs was performed using ClustalX 1.83^4^, and phylogenetic tree construction was performed with MEGA 6.0 based on the neighbor-joining (NJ) sequence distance method (Tamura et al., 2013). Amino acid sequence translation was performed using DNAMAN, and secondary structure prediction was performed using SOPMA^5^.

### Generation of Transgenic *Arabidopsis* and *Nicotiana tabacum*

The overexpression vector was constructed using the binary vector pBI121 containing a cauliflower mosaic virus 35S (CaMV35S) promoter and NOS terminator (Wang et al., 2017). Amplification of the coding sequence of *LcNAC13* including the restriction enzyme cut sites was performed using pBI121-LcNAC13-F/R primers (Supplementary Table 1). Double digestion of the pBI121 plasmid was performed with *Xba*1 and *Sma*1 restriction endonucleases. The target fragment was inserted behind the CaMV35S promoter using the In-Fusion^®^HD Cloning Kit. *Agrobacterium* strain GV3101 cells containing the pBI121-LcNAC13 recombinant plasmid were infested with Arabidopsis flower clusters using the *Agrobacterium*-mediated transformation system (Senthil-Kumar and Mysore, 2014; Zhang et al., 2006). Kanamycin-resistant transgenic seedlings were identified by PCR using primers 35S:LcNAC13-F/R and *NptIIu*-F/R (Supplementary Table 1).

For tobacco transformation, we used the binary vector pBI121-LUC (Wang et al., 2017). The pBI121-LUC was double-digested by *Xba*1 and *Bam*HI. The target fragment of *LcNAC13* was amplified using primer pBI121:LcNAC13:LUC-F/R (Supplementary Table 1). The target fragment was inserted into the pBI121-LUC expression vector following the CaMV35S promoter using the In-Fusion^®^HD Cloning Kit. The successfully constructed vector was transformed into *Agrobacterium* strain GV3101. *N. tabacum* transformation was carried out according to the method of Ding et al. (2015).

### Luciferase Imaging

Leaves were first sprayed with 1 mM D-luciferin potassium (Goldbio, United States) and 0.01% Triton X-100. After being placed in the dark for 20 min, luciferase (LUC) activity expressed by bioluminescence intensity was measured using a deep-cooled CCD imager (AndoriXon; Andor) and Meta Imaging Service software (Meta Vue Version 7.8.0.0). LUC fluorescence signal is detected to indicate the successful expression of the exogenous gene in the plant, and the intensity of the fluorescence can reflect the level of target gene expression.

### Virus-Induced Gene Silencing of *LcNAC13*

For silencing the *LcNAC13*, we used tobacco rattle virus (TRV)-derived vectors, provided by Prof. Qin-Long Zhu from Key Laboratory of Innovation and Utilization for Germplasm Resources in South China Agricultural University. A 327-bp fragment of *LcNAC13* including the restriction enzyme cutting site was amplified from pTRV2*-LcNAC13*-F/R (Supplementary Table 1). The pTRV2 vector was double-digested by *Xba*I and *Sma*I. The target fragment was inserted into the vector using the In-Fusion^®^HD Cloning Kit. VIGS was carried out according to the method of Schachtsiek et al. (2019) and Shi et al. (2021). The successfully transformed GV3101 strain containing TRV-VIGS vector was placed in a YEP medium containing 10 mM MES, 20 mM acetosyringone, 25 μg/ml rifampicin, and 50 μg/ml kanamycin and incubated at 28°C for 24 h. *Agrobacterium* cells were collected by centrifugation and added to an infiltration buffer (OD600 = 1-2) containing 200 mM acetosyringone, 10 mM MgCl_2_, and 10 mM MES (pH 5.6). *Agrobacterium* cultures containing pTRV1 and pTRV2 (control) and pTRV1- and pTRV2-Lc*NAC13* were mixed at a ratio of 1:1 (v/v) and placed in the dark for 4 to 6 h before inoculation of plants. The mixture was injected into the base of the stem of new litchi shoots using a 1-ml syringe. Two days after infection, 120 μM of MV was sprayed on the injected shoots to accelerate the rudimentary leaf senescence. Leaves were collected at 0, 30, and 60 h after MV treatment for qRT-PCR analysis, and those at 30 h were collected for library construction. The percentage of the yellowish leaf was determined after 0, 1, 3, 5, and 7 days of 120 μM MV treatments. Leaflets with one-third of the surface turned brown were defined as senescent leaves. The percentage of the brown leaf was calculated as the percentage of brown leaflets to total leaflets in one shoot (Wang et al., 2017).

### RNA Extraction, Library Construction, and RNA Sequencing of Virus-Induced Gene Silencing Samples

We used the Plant Total RNA Isolation Kit (Huayueyang, Beijing, China) for total RNA extraction and Oligo-dT beads (Qiagen, Valencia, United States) for mRNA enrichment. RNA was fragmented into short fragments by fragmentation buffer and reverse-transcribed into cDNA by random primers. Second-strand cDNA was synthesized by DNA polymerase I, RNase H, dNTPs, and buffer. We then used Qiaquick PCR Extraction Kit (Qiagen, United States) for the purification of cDNA fragments. The fragments were end-repaired, poly (A) added, and ligated to Illumina sequencing adapters. The size-selected fragments were amplified and sequenced by Illumina HiSeqTM 4000 by Gene *Denovo* Biotechnology Co. (China). A 125 paired-end (PE) module was used. Before library construction, all the VIGS-treated leaves were subjected to qRT-PCR analysis. Only those whose target gene expression decreased up to twice were used. Then, six libraries representing three biological replicates for treatment and control were constructed.

### RNA-Seq Data Analysis of Virus-Induced Gene Silencing

Adapters in the raw reads were filtered. Reads with more than 10% of unknown nucleotides and with over 50% low *Q*-value (≤10) bases were removed. To construct reference sequences, clean reads in all the samples were *de novo* assembled by the Trinity (Version 2.0) Program. The high-quality clean reads were mapped to ribosome RNA (rRNA) to identify residual rRNA reads. The rest of the reads were subjected to further analysis.

Unigenes annotation in the data was performed using the BLASTx program (see text footnote 2) with an *E*-value threshold of 1e^–5^ and compared to the NCBI nr database, Swiss-Prot protein database, KEGG database, and COG database, respectively. The best comparison information was selected as the annotation results of unigenes. In case of inconsistent information between different databases, the selection was made in the order of nr, Swiss-Prot, KEGG, and COG. When a unigene does not match any information, the sequence orientation will be confirmed by the EST scan program. GO functional annotation was performed by Blast2GO software, and unigene functional classification was performed using WEGO software. KEGG pathway annotation was performed by BlastX software. The dataset is available in the NCBI SRA under the accession number SRP162301.

Clean reads of one sample were uniformly mapped into the reference sequence database by Bowtie2 software. The number of unique-match reads was calculated and normalized to RPKM (reads per kb per million reads) for gene expression analysis. A comparison of unigene expression between treatments and controls was made by the *DESeq* (Anders and Huber, 2010)^6^ with FDR ≤0.05 and the absolute value of log_2_ ratio ≥1.

### Statistical Analysis

Statistical package for the social sciences program (SPSS, Chicago, IL, United States) was used to analyze variances. The differences among treatment means were evaluated by Duncan’s multiple range test at 0.05 and 0.01 probability levels. The differences between the treatment and the control were evaluated by Student’s *t*-test at 0.05 and 0.01 probability levels.

To explore the relationships between differentially expressed genes (DEGs) in the transcriptome of VIGS samples, a hypothetical model was constructed by PLS-SEM with SmartPLS 2.0 M3 software (Ringle et al., 2014; Chen et al., 2017). The standardized path coefficient values were calculated with the PLS algorithm by Path Weighting Scheme using a bootstrapping method to get the significance of path coefficients. The sign changes were individual. The samples during the calculation of bootstrapping method were 5000. In the PLS model, differentially expressed hormonal regulation-related genes of IAA, JA, GA, ABA, and CTK were screened for modeling. In addition, the three top numbers of the identified TF-encoding genes, namely, *NAC*s, *MYB*s, and *WRKY*s, were also selected as the group for model building.

## Results

### Low Temperature Induces Panicle Leaf Senescence and Promotes Floral Development

**FIGURE 1:**
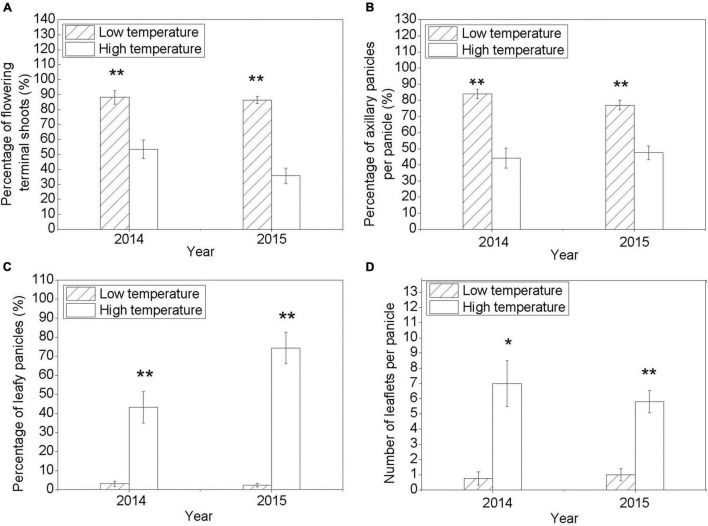
Flowering phenotype of the litchi trees under low temperature (LT) and high temperature (HT) conditions. **(A)** Percentage of the flowering terminal shoots. **(B)** Percentage of the axillary panicles per panicle. **(C)** Percentage of leafy panicles. **(D)** Number of leaflets per panicle. Vertical bars represent standard error (SE; *n* = 3). Symbols above the column mean a significant difference between LT and HT treatments (Duncan’s multiple range test, *P = 0.05; **P = 0.01).

**FIGURE 2:**
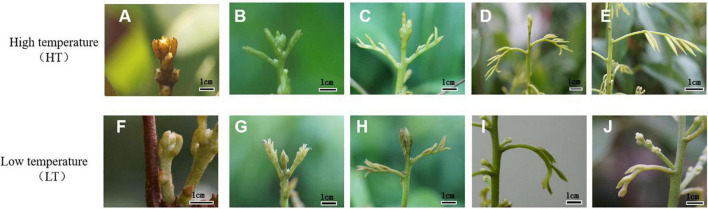
Morphology of litchi rudimentary leaves and flower buds under HT and LT conditions. **(A–E)** Different developmental stages of the leaf on panicle and flower buds under HT condition. **(F–J)** Different developmental stages of the leaf on panicle and flower buds under LT condition.

As shown in Figure 1, the percentage of the flowering terminal shoots and the percentage of the axillary panicles per panicle under LT were significantly higher than those under HT condition. However, the percentage of leafy panicles and the number of leaflets per panicle under HT condition were higher than those under LT condition. The results suggested that LT induced rudimentary leaf senescence and promoted floral development in litchi. The developmental state of the rudimentary leaves was divided into five stages. The morphology of the senescing leaves under LT condition and that of the developing leaves under HT condition are shown in Figure 2. Under LT condition, the leaves on the panicle could not develop normally and become prematurely senescent, while the flower buds could develop. Under HT condition, the leaves in the panicle developed, while the flower buds aborted, suggesting that the growing rudimentary leaves might affect the development of flower buds.

### Identification of *NACs* Potentially Involved in Reactive Oxygen Species-Induced Rudimentary Leaf Senescence

We analyzed a previous RNA-Seq dataset of the MV-treated rudimentary leaves and identified 659 *NACs* (Supplementary Table 2). Fourteen out of the *NACs* show upregulated expression trends at 0, 5, and 10 h of ROS treatment. Among them, expression levels of *LcNAC13* increased with the time of ROS treatments (Supplementary Figure 1) and with the senescence (Lu et al., 2014). Therefore, this *LcNAC13* gene was further investigated.

### *LcNAC13* Is Highly Expressed During Low-Temperature- and Reactive Oxygen Species-Induced Rudimentary Leaf Senescence

**FIGURE 3:**
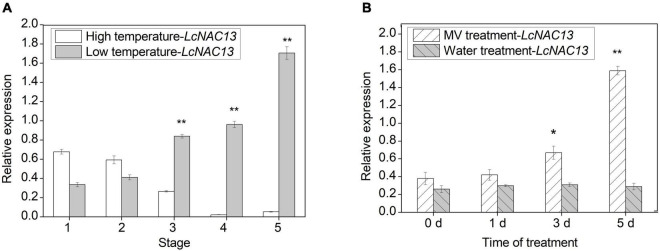
Expression of *LcNAC13* in response to temperature **(A)** and reactive oxygen species (ROS) **(B)** treatments. Vertical bars represent SE(*n* = 3); an asterisk indicates a significant difference according to Student’s *t*-test (*P* ≤ 0.05) and double asterisks indicate a significant difference according to Student’s *t*-test (*P* ≤ 0.01).

To investigate the expression of *LcNAC13* in response to LT and HT conditions, the relative expression of *LcNAC13* at different stages as shown in Figure 3 was determined. The results showed that the expression of *LcNAC13* under LT condition increased from stage 1 to stage 5, while those under HT condition slightly decreased (Figure 3A). The expression of *LcNAC13* in response to ROS was also studied. As shown in Figure 3B, the expression of *LcNAC13* in the ROS-treated rudimentary leaves showed an increasing trend from 0 to 5 days of treatment. Expression levels of the *LcNAC13* in the ROS-treated leaves were significantly higher than those of the control at 3 and 5 days of treatment. Taking together, *LcNAC13* may play an important role in the LT- and ROS-induced senescence of the rudimentary leaves.

### Cloning and Characterization of *LcNAC13*

The full-length sequence of *LcNAC13* in litchi was obtained by cloning with specific primers (Supplementary Table 1). We identified a 1101-bp-long open reading frame (ORF) encoding a protein of 367 deduced amino acids. The cDNA sequence of *LcNAC13*, including 5′ and 3′ UTR, is shown in Supplementary Figure 2. The NAC domain was originally characterized from consensus sequences from petunia NAM and from Arabidopsis ATAF1, ATAF2, and CUC2. As shown in Supplementary Figure 3, the deduced LcNAC13 protein contains a highly conserved region in its N-terminal sequence that may function as a DNA-binding domain. The N-terminal residues contain five subdomains (A–E) (Supplementary Figure 3). The C terminus of LcNAC13 protein, serving as a transcription activation domain, shows low sequence similarity to other *Arabidopsis* NAC proteins.

**FIGURE 4:**
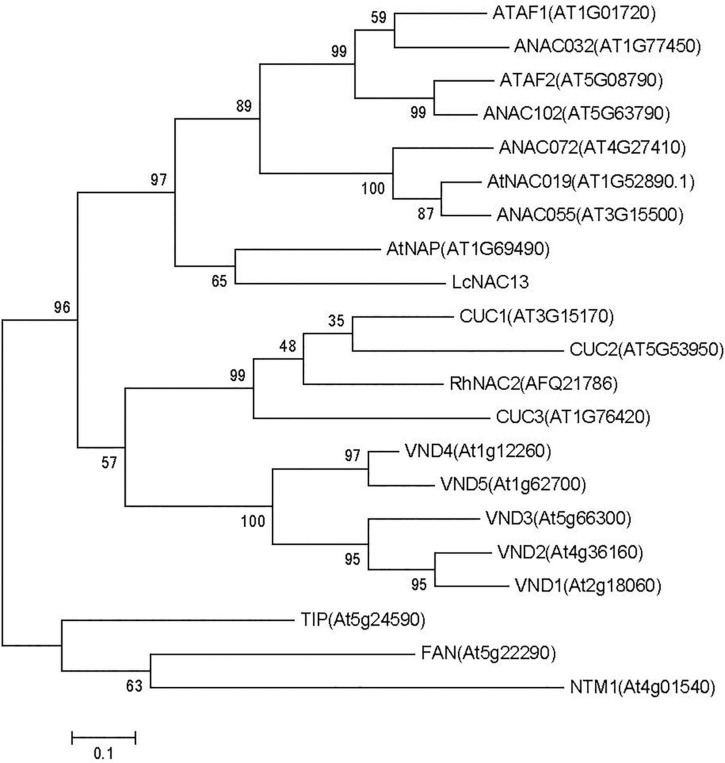
Phylogenetic tree analysis of NAC. From the results of the evolutionary tree analysis, the most similar one to LcNAC13 is AtNAP. A phylogenetic tree was constructed by neighbor-joining (NJ) method using MEGA 6.0 program.

A phylogenetic tree for the LcNAC13 domain and typical Arabidopsis NAC family proteins is shown in Figure 4. NAC domains were classified into two large groups, and the LcNAC13 proteins fell into Group I. In addition, the NAC domains in each group could be divided into several subgroups according to the similarity of the NAC-domain structures. Noticeably, NACs with the same functions showed a tendency to fall into one subgroup. For instance, LcNAC13 and AtNAP were clustered into one subgroup, suggesting that they may have the same function. *AtNAP* belongs to the NAC family of TFs, and the expression of this gene is associated with senescence in *Arabidopsis*. Inducible overexpression of *AtNAP* leads to premature leaf senescence (Luoni et al., 2019).

### Overexpression of *LcNAC13* Promotes Reactive Oxygen Species-Dependent and Natural Senescence in *Arabidopsis* and Tobacco

**FIGURE 5:**
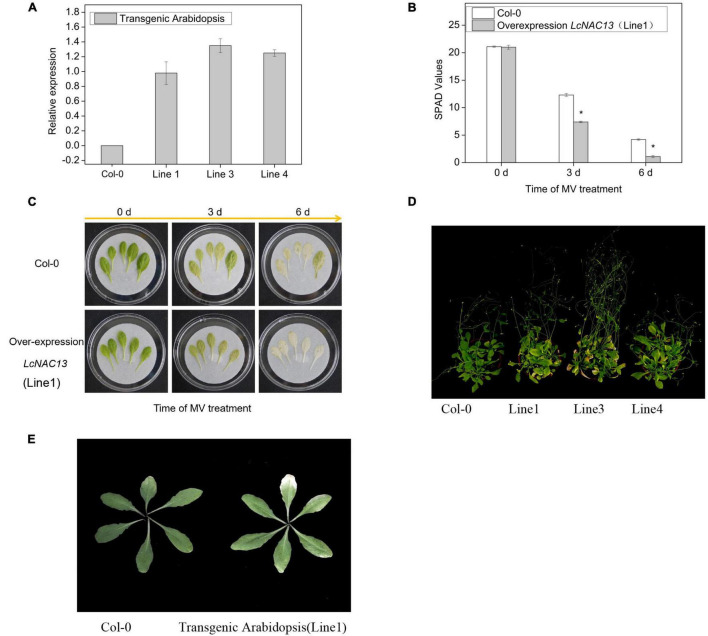
Overexpression of *LcNAC13* promotes ROS-dependent and nature senescence in *Arabidopsis thaliana.* **(A)** Relative expression of *LcNAC13* in the three transgenic lines. **(B)** SPAD values in leaves of the transgenic and wild-type plants after methyl viologen dichloride hydrate (MV) treatment. **(C)** Phenotypic changes in leaves of the transgenic and the wild-type plants after MV treatment. **(D)** Phenotype of the transgenic and the wild-type plants showing natural senescence. **(E)** Rosette leaves of the transgenic and wild-type plants 32 days after planting. Vertical bars represent SE (*n* = 3); an asterisk indicates a significant difference according to Student’s *t*-test (*P* ≤ 0.05).

To analyze the function of *LcNAC13*, we performed heterologous genetic transformation of *A. thaliana* and tobacco. As shown in Figure 5A, the expression of *LcNAC13* significantly increased in the transgenic *Arabidopsis* plants. As one of the symptoms of ROS-dependent senescence is the decrease in leaf chlorophyll (Wang et al., 2017), the SPAD values corresponding to the amount of chlorophyll in the leaves were determined. As shown in Figure 5B, after 3 and 6 days of MV treatment, the leaf SPAD values in the *LcNAC13* transgenic plants were significantly lower than those in the wild-type ones, respectively. The green color of the transgenic leaves faded faster than that of the wild-type leaves (Figure 5C). Moreover, the natural senescence of the transgenic plants was accelerated (Figures 5D,E). These results suggested that overexpression of *LcNAC13* promoted ROS-dependent and natural senescence in *Arabidopsis*.

**FIGURE 6:**
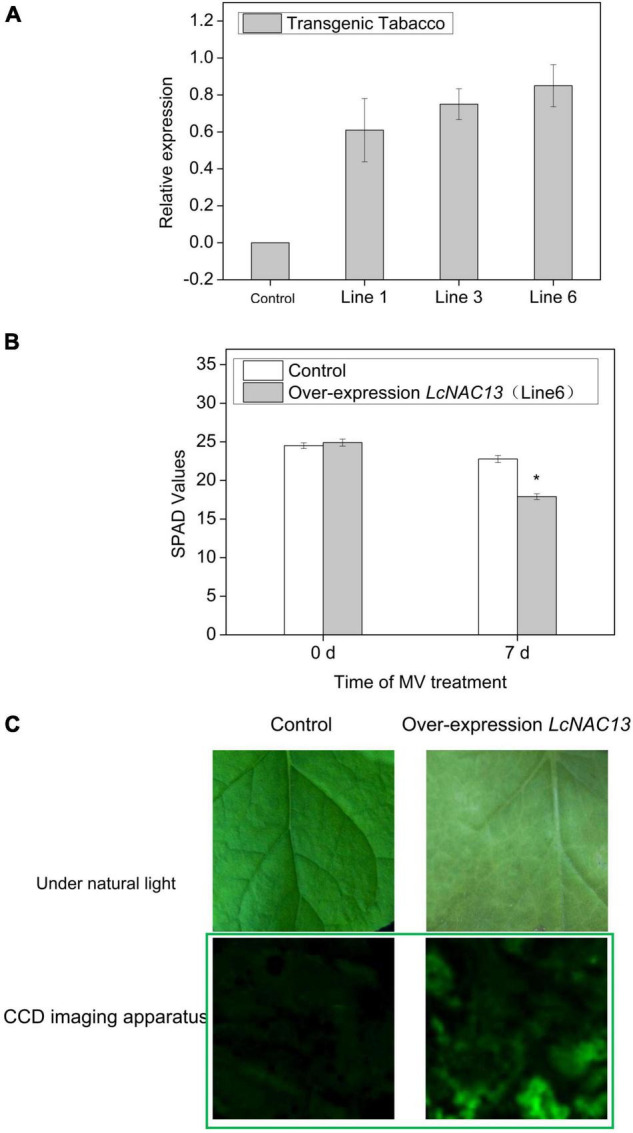
Overexpression of *LcNAC13* promotes ROS-dependent senescence in tobacco. **(A)** Relative expression of *LcNAC13* in the transgenic and wild-type plants. **(B)** SPAD values in leaves of the transgenic and wild-type plants after MV treatment. **(C)** Phenotype of the *LcNAC13* transgenic tobacco leaves after 10 days of MV treatment. Vertical bars represent SE (*n* = 3); an asterisk indicates a significant difference according to Student’s *t*-test (*P* ≤ 0.05). The green box indicates imaging under a deep-cooled CCD imager.

For overexpression of *LcNAC13* in tobacco, we used a firefly luciferase (LUC) reporter. As shown in Figure 6, overexpression of *LcNAC13* in tobacco reduced the SPAD values after ROS treatment. Bioluminescence indicating the reporter could be detected in the leaves of the transgenic plants. Here, we used LUC as a marker to observe the expression of the target gene of *LcNAC13*. The senescence phenotype was judged by the SPAD values. The results further suggest that the enhanced expression of *LcNAC13* promoted leaf senescence.

### Silencing *LcNAC13* Delays Reactive Oxygen Species-Dependent Senescence and Changes the Gene Expression Patterns of the Rudimentary Leaves

**FIGURE 7:**
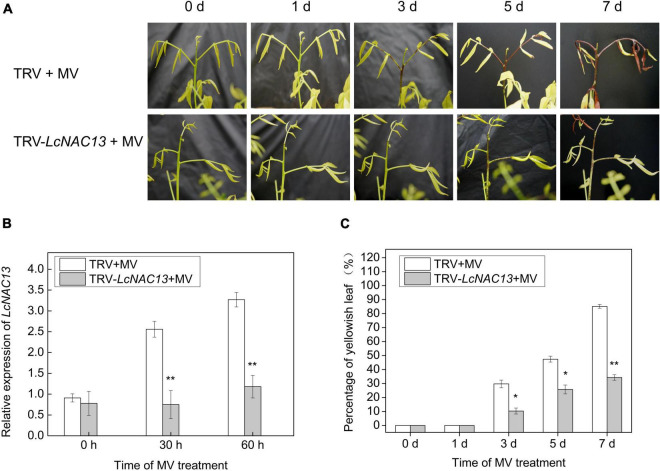
*LcNAC13* silencing delays MV-induced senescence in litchi leaves. **(A)** Phenotype of the virus-induced gene silencing- (VIGS) treated and control leaves after MV-generated ROS treatment. TRV + MV represents control, and TRV–*LcNAC5* + MV represents VIGS treatment. **(B)** *LcNAC13* expression level of the VIGS-treated and control leaves after MV treatment. **(C)** Percentage of the yellowish leaves. Data are means of three replicates, and the bars represent SEs. Significant differences according to the Student’s *t*-test between VIGS-treated and control leaves at the same time point are indicated by the asterisks (*P < 0.05, **P < 0.01).

Virus-induced gene silencing experiments were carried out for further functional study on *LcNAC13*. Meanwhile, based on the transcriptome sequencing data of the silencing samples and the control samples, it is possible to understand the changes in the expression pattern of genes regulated by *LcNAC13*. As shown in Figure 7, after 3 days of MV treatment, rudimentary leaves infected with pTRV2-*NAC13* delayed the browning compared with the TRV controls. qRT-PCR analysis showed that *LcNAC13* expression in the TRV2-*LcNAC13*-treated leaves was significantly downregulated at 30 and 60 h of infection (Figure 7B). Accordingly, after 3, 5, and 7 days of MV-generated ROS treatment, the percentage of yellowish leaf after silencing *LcNAC13* was twofold, onefold, and 1.5-fold lower than the control, respectively (Figure 7C). These results suggest that silencing *LcNAC13* delayed ROS-dependent senescence in litchi leaves.

### Digital Transcriptome Analysis and Identification of Genes Involved in Silence of *LcNAC13*

As decreased *LcNAC13* expression was found at 30 h of ROS treatment, samples at 30 h after silencing treatment were selected for RNA-Seq. Six RNA-Seq libraries of the control and TRV-*NAC* (silenced samples) were constructed to identify genes regulated by *LcNAC13*. Compared with the control leaves, silencing *LcNAC13* resulted in 1857 significantly upregulated and 733 significantly downregulated DEGs (Supplementary Figure 4).

**FIGURE 8:**
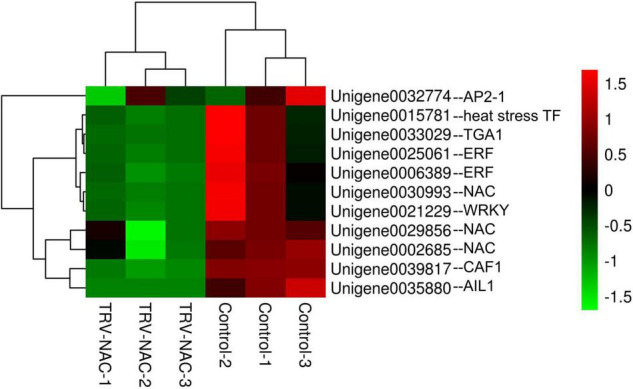
Heatmap analysis of the downregulated transcription factor (TF) in GO:0034645. Data were normalized to *Z*-score.

GO-term analysis was performed among the DEGs. From the enriched GO term including regulation of cellular macromolecule biosynthetic process, ROS metabolic process, developmental process, carbohydrate metabolic process, ethylene metabolic process(Supplementary Table 3), we identified 11 DEGs (Figure 8), including *LcNAC13*. They encode homologous proteins including three ethylene-responsive TFs (ERF), three NAC TFs (NAC), one WRKY transcription factor (WRKY), one TF TGA1-like (TGA1), one TF APETALA2 isoform 1 (AP2-1), one probable CCR4-associated factor 1 (CAF1), and one heat stress TF family protein (Figure 8).

### Identification of Differentially Expressed Transcription Factors Involved in Silence of *LcNAC13*

By screening differentially expressed TF-encoding genes, a total of 691 of the TFs including 163 downregulated TFs and 528 upregulated TF-encoding genes were screened. *BHLH*, *NAC*, *HSF*, and *MYB* were the most significantly downregulated genes (Supplementary Figure 5). These TFs might be regulated by *LcNAC13.*

### Pathway Involved in the Silence of *LcNAC13*

To reveal a framework of the DEGs of *LcNAC13*-silenced leaves, we performed PLS-SEM analysis using the transcriptome data of the *LcNAC13*-related enrichment function (GO) and metabolic pathway (KEGG) and significant downregulation of TFs including NACs, WRKYs, and MYBs. The use of PLS methods for data analysis is due to the fact that PLS is not only good at eliminating covariance between variables, but also stable in working across multiple databases. Most of the latent variables were significant when analyzed in PLS-SEM, and the external model block could provide an adequate explanation of the latent variables (Supplementary Table 4). In this calculated model, when the values of average variance extracted (AVE, an indicator for converge validity) and composite reliability (indicator for internal consistency reliability) are higher than 0.5 and 0.7, respectively, (Supplementary Table 5), it means that the model is available (Hair et al., 2014).

**FIGURE 9:**
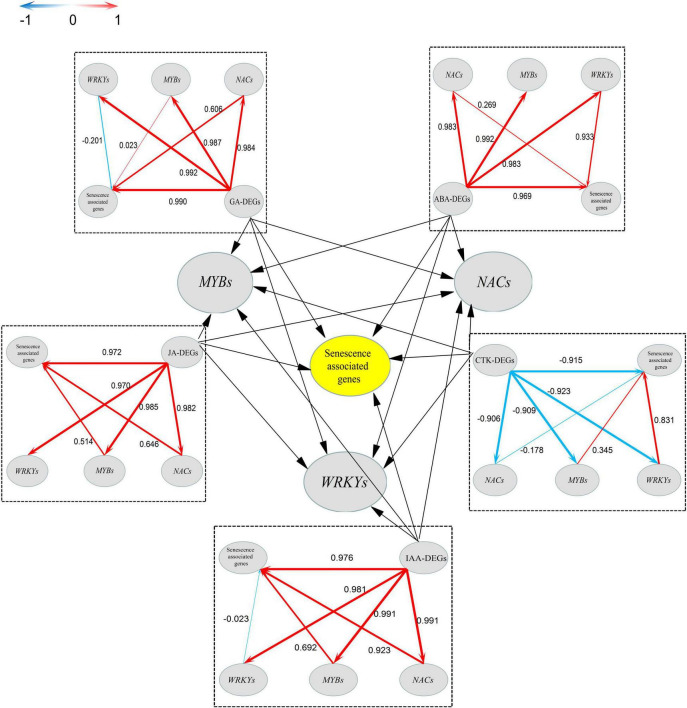
Direct diagram of the partial least squares structural equation model (PLS-SEM). The central part of the plot is based on the other five surrounding parts. The level of the path coefficients is reflected by the width of the red (positive effects) or blue (negative effects) arrows. The values on the arrows indicate the intensity of factor loading for that pathway. The pathways from hormone-related differentially expressed genes (DEGs) such as ABA, IAA, CTK, JA, and GA to TFs are first order for DEGs encoded by TFs, while the pathway from DEGs encoded by TFs to senescence-associated genes (SAGs) is second order.

As shown in Figure 9, there are two forms of direct pathways and indirect pathways. Among the three classes of significant downregulated expression TFs, positive or negative associations were established with DEGs associated with the senescence-associated genes (SAGs), respectively. In the ABA, IAA, GA, and JA hormone modules, ABA-DEGs, IAA-DEGs, GA-DEGs, and JA-DEGs were correlated with the SAGs directly with a factor loading above 0.96, suggesting a strong influence relationship in these backgrounds. CTK-DEGs with SAGs had a high factor loading in the CTK model (–0.915), and the absolute value of it is higher than the second-order indirect pathways. However, the absolute value of factor loading of all DEGs with *NACs*, *WRKYs*, and *MYBs* was greater than 0.96, suggesting a strong influence relationship between hormone DEGs and TFs in these backgrounds.

**FIGURE 10:**
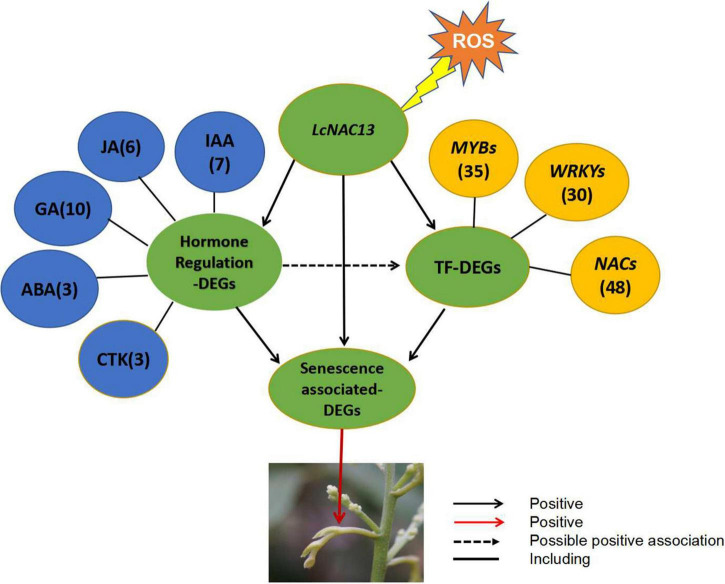
A proposed model for the ROS-induced rudimentary leaf senescence regulated by *LcNAC13.* The numerals in the parentheses represent the number of differentially expressed genes. The relationship of the groups of the DEGs is shown in Figure 9. The genes information inside the mapping is shown in Supplementary Figure 6.

According to the PLS model, we proposed a model for the ROS-induced rudimentary leaf senescence regulated by *LcNAC13* (Figure 10). *LcNAC13* may directly regulate the expression of SAGs. It may also regulate the expression of hormonal regulation-related genes and the TF-encoding genes, namely, *MYB*s, *WRKY*s, and *NAC*s. Then, these genes may affect the expression of the SAGs. Moreover, *LcNAC13* may indirectly affect the expression of *MYB*s, *WRKY*s, and *NAC*s through hormonal regulation-related genes. Finally, the changed expressions of the SAGs resulted in rudimentary leaf senescence.

## Discussion

NAC transcription factors are widely found in plants. Several *NAC* genes related to plant senescence have been verified (Zhou et al., 2013; Trupkin et al., 2019; Ma et al., 2021). In our previous sequencing analysis, members of the NAC family related to the senescence of rudimentary leaves in litchi were identified and the MV-generated ROS was used to induce the senescence of rudimentary leaves in litchi (Liu et al., 2013; Lu et al., 2014). MV can generate superoxide by accepting an electron from PS1 (Dodge, 1971). In this study, we showed that *LcNAC13* was involved in the ROS-induced and LT-induced senescence of litchi rudimentary leaves. Senescence of the leaves in Arabidopsis and tobacco was accelerated by *LcNAC13*. Previous studies showed that the NAC TF is involved in the control of seed development, abiotic stress resistance, and disease resistance (Puranik et al., 2012; Forlani et al., 2021; Yuan et al., 2021). NAP belonging to the NAC family can regulate plant growth and the leaf apoptosis process (Guo and Gan, 2006; Greco et al., 2012; Fan et al., 2015). Our study showed that *LcNAC13* was involved in leaf senescence in litchi. The phylogenetic tree indicated that *LcNAC13* in litchi had the highest similarity with NAP in *Arabidopsis*. Meanwhile, NAP is well known as a central factor in the regulation of plant senescence (Luoni et al., 2019; Zou et al., 2021). Hence, it is likely that *LcNAC13* may be an important factor regulating senescence in litchi leaves.

Our comprehensive analysis of the VIGS-*LcNAC13* sequencing data indicated that the silence of *LcNAC13* may cause a series of changes, including metabolic process and cell and catalytic activity according to the GO enrichment analysis. According to the KEGG enrichment analysis, the synthesis of starch and sugar, the synthesis of phenylpropanoid, and photosynthesis were also changed. The results suggested that *LcNAC13* might control leaf senescence by altering a series of processes.

Plant hormones are important regulators involved in plant growth and development. The plant hormone signals can be transmitted to the nucleus through a series of signal transduction components. As a result, these signals can activate gene expression and alter the physiological processes (McSteen and Zhao, 2008; Qu and Zhao, 2011; Song et al., 2017). TFs can bind to specific DNA sequences as a way to control the transcription and expression of specific genes (Spitz and Furlong, 2012; Todeschini et al., 2014; Rastogi et al., 2018). In plants, NAC, WRKY, and MYB TFs are central TFs for hormonal regulation and often play important regulatory roles in growth and development (Xu et al., 2019; Fraga et al., 2021; Wu et al., 2021). In order to obtain directly information related to the *LcNAC13*-controlled senescence in the rudimentary leaves, we established an *LcNAC1*3-silencing system using VIGS. We then performed RNA-Seq of the leaf samples. In the VIGS-*LcNAC13* sequencing data, differentially downregulated expression genes were screened including a number of *NAC*s, *WRK*Ys, *MYB*s, ABA-DEGs, IAA-DEGs, JA-DEGs, and rudimentary leaf senescence-associated DEGs. Interestingly, by the analysis of PLS-SEM, we found that ABA-, auxin-, JA-, and GA-related DEGs all contributed directly and positively to rudimentary leaf senescence-related DEGs. Only the CTK module was negatively contributed to leaf senescence-related genes (Figure 10 and Supplementary Table 6). In the analysis of PLS-SEM, we can use the factor loading value to distinguish which pathways are more influential between the direct and indirect pathways. Comprehensive data analysis suggests PLS-SEM is valuable in revealing relationships between biological processes based on RNA-Seq data (Liu et al., 2019).

## Conclusion

In this study, the *LcNAC13* was isolated and characterized. The results of gene expression analysis showed that *LcNAC13* was involved in both LT- and ROS-promoted rudimentary leaf senescence in litchi. The highest similarity between LcNAC13 and AtNAP was found by constructing a phylogenetic tree analysis. Transformation of *LcNAC13* in *Arabidopsis* and tobacco accelerated leaf senescence. Silencing *LcNAC13* delayed the ROS-dependent senescence and changed the gene expression patterns of the rudimentary leaves. The results of our study suggested that *LcNAC13* might be a key factor in promoting rudimentary leaf senescence through different signaling pathways. Our work provided an important target gene for the future molecular breeding of new cultivars that could flower in warmer climates.

## Data Availability Statement

The datasets presented in this study can be found in online repositories. The names of the repository/repositories and accession number(s) can be found below: https://www.ncbi.nlm.nih.gov/, SAR158542; https://www.ncbi.nlm.nih.gov/, SRP162301.

## Author Contributions

BZ contributed to the design of the research and was contributor in writing the manuscript. CW performed sample collection, gene expression analysis, and transformation and was contributed in writing the manuscript. HL and LH analyzed the transcriptome data. HC and XL participated in the design of the research. All authors read and approved the manuscript.

## Conflict of Interest

The authors declare that the research was conducted in the absence of any commercial or financial relationships that could be construed as a potential conflict of interest.

## Publisher’s Note

All claims expressed in this article are solely those of the authors and do not necessarily represent those of their affiliated organizations, or those of the publisher, the editors and the reviewers. Any product that may be evaluated in this article, or claim that may be made by its manufacturer, is not guaranteed or endorsed by the publisher.

## Footnotes

1
http://www.ncbi.nlm.nih/sra

2
http://www.ncbi.nlm.nih.gov/BLAST/

3
http://www.ncbi.nlm.nih.gov/Structure/cdd/wrpsb.cgi

4
http://www.ebi.ac.uk

5
https://npsa-prabi.ibcp.fr/cgi-bin/npsa_automat.pl?page=npsa_sopma.html

6
http://bioconductor.org/packages/release/bioc/html/DESeq2.html

## Supplementary Material

Supplementary Figure 1 Heatmap diagram showing the expression profiles of the *NACs* in the rudimentary leaves. RPKM (reads per kb per million reads) values of the samples were normalized to *Z*-score.

Supplementary Figure 2 Full-length sequence and amino acid translation of *LcNAC13.*

Supplementary Figure 3 Sequence alignment of LcNAC13 protein with typical *Arabidopsis* NAC-domain proteins. Sequence alignment of the quantified sequences of the NAC domains in their respective groups and subgroups. Subdomains A to E are shown by arrows above the sequences. Amino acids in the consensus sequences that are common to all groups are shown in dark blue (=100%). Amino acids in the consensus sequences with identity over 80% are shaded in red, whereas those with identity between 50 and 75% are shaded in light blue.

Supplementary Figure 4 Analysis of the differentially expressed genes (DEGs) between control and TRV-NAC (the silenced samples). **(A)** Each dot represents a gene. The *x*-axis represents the mean expression of the compared genes. The *y*-axis is the logarithm with base 2 of the fold change of the compared genes. Red dots represent DEGs. **(B)** Statistics of DEGs between control and TRV-NAC samples.

Supplementary Figure 5 Classification of transcription factors (TFs) in differentially expressed genes.

Supplementary Table 6 Genes information in Figure 10.
