# Supplementary figures and images for: LcNAC13 Is Involved in the Reactive Oxygen Species-Dependent Senescence of the Rudimentary Leaves in Litchi chinensis
Source: Front Plant Sci. 2022 May 9;13:886131. doi: 10.3389/fpls.2022.886131 (PMC9125249; doi:10.3389/fpls.2022.886131)

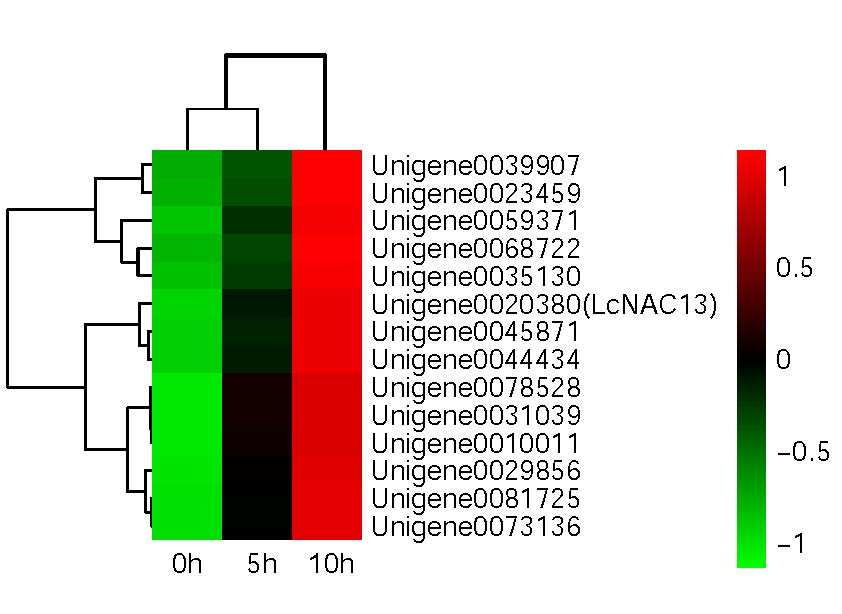

Supplement: Supplementary Figure 1 — Heatmap diagram showing the expression profiles of the NACs in the rudimentary leaves. RPKM (reads per kb per million reads) values of the samples were normalized to Z-score. [file Image_1.JPEG]

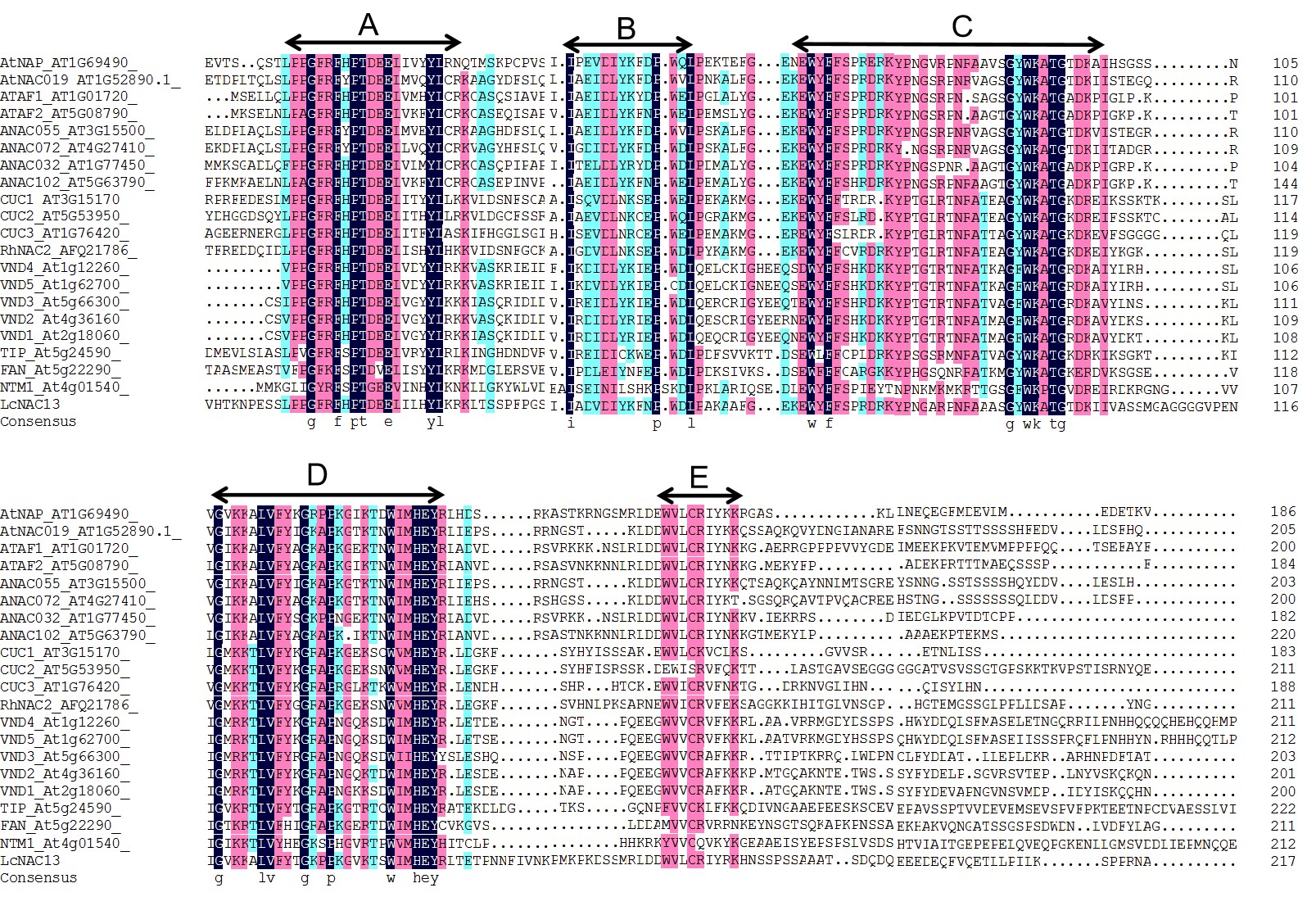

Supplement: Supplementary Figure 2 — Full-length sequence and amino acid translation of LcNAC13. [file Image_2.JPEG]

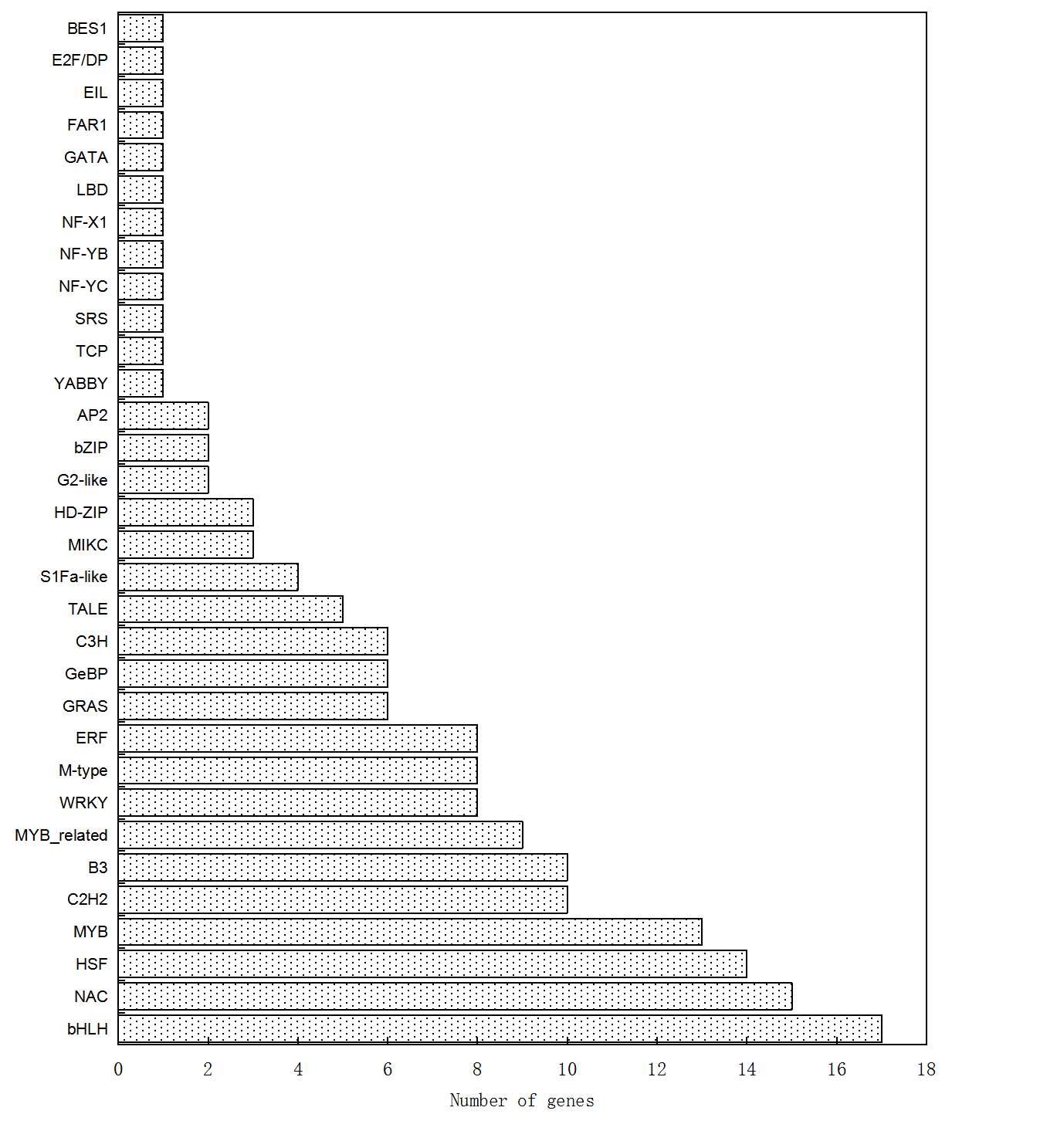

Supplement: Supplementary Figure 3 — Sequence alignment of LcNAC13 protein with typical Arabidopsis NAC-domain proteins. Sequence alignment of the quantified sequences of the NAC domains in their respective groups and subgroups. Subdomains A to E are shown by arrows above the sequences. Amino acids in the consensus sequences that are common to all groups are shown in dark blue (=100%). Amino acids in the consensus sequences with identity over 80% are shaded in red, whereas those with identity between 50 and 75% are shaded in light blue. [file Image_3.JPEG]

**A**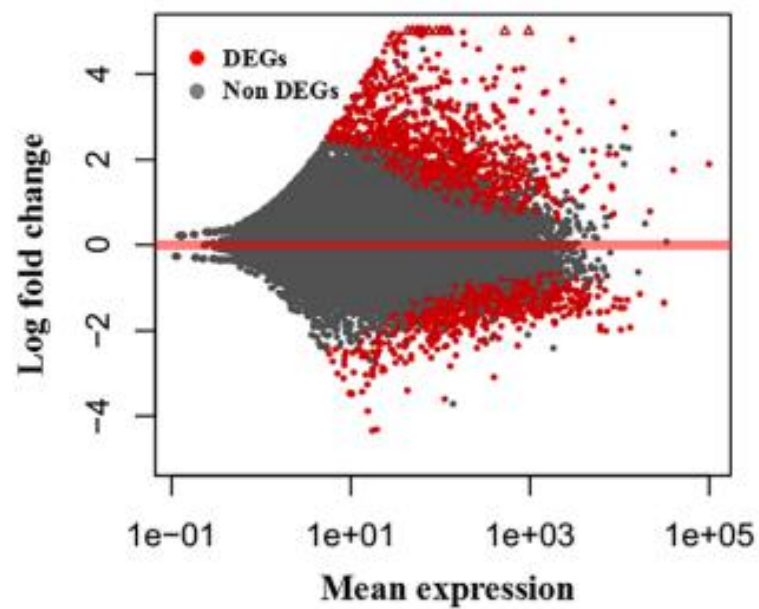**B**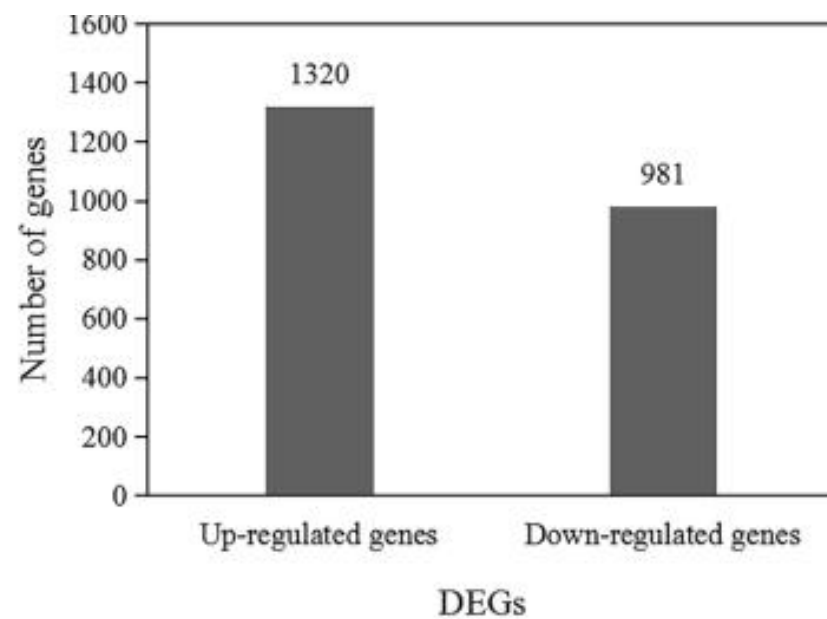

Supplement: Supplementary Figure 5 — Classification of transcription factors (TFs) in differentially expressed genes. [file Data_Sheet_2.PDF]
