# Supplementary material for: LcNAC13 Is Involved in the Reactive Oxygen Species-Dependent Senescence of the Rudimentary Leaves in Litchi chinensis
Source: Front Plant Sci. 2022 May 9;13:886131. doi: 10.3389/fpls.2022.886131 (PMC9125249; doi:10.3389/fpls.2022.886131)
Supplement: Supplementary Figure 4 — Analysis of the differentially expressed genes (DEGs) between control and TRV-NAC (the silenced samples). (A) Each dot represents a gene. The x-axis represents the mean expression of the compared genes. The y-axis is the logarithm with base 2 of the fold change of the compared genes. Red dots represent DEGs. (B) Statistics of DEGs between control and TRV-NAC samples. [file Data_Sheet_1.PDF]

	5’ UTR
	CCTTCTTGTCTTCTCTCTTCGCTCTCTTGCTTTTCAATTGCAAGACAGTTTGTTTCTTTT
	CTTTTTTTTCTACTAGTC
1	ATGGTTCACACGAAGAACCCTGAATCATCCCTGCCTCCAGGGTTCAGGTTCCACCCTACT
1	M V H T K N P E S S L P P G F R F H P T
61	GATGAGGAACTCATCCTTCATTATCTCAAGAGGAAGCTTACTTCTTCACCTTTCCCAGGT
21	D E E L I L H Y L K R K L T S S P F P G
121	TCCATCATTGCTGATGTTGATATCTACAAGTTTAATCCTTGGGACTTACCAGCTAAAGCA
41	S I I A D V D I Y K F N P W D L P A K A
181	GCTTTTGGTGAGAAAGAGTGGTACTTTTTCAGTCCTAGAGACCGAAAGTACCCGAATGGA
61	A F G E K E W Y F F S P R D R K Y P N G
241	GCCAGGCCAAACAGGGCAGCTGCATCAGGGTATTGGAAGGCAACTGGCACGGATAAGATC
81	A R P N R A A A S G Y W K A T G T D K I
301	ATAGTAGCATCTTCAATGGGAGCAGGAGGTGGTGGTGTGCCAGAGAACATTGGTGTCAAA
101	I V A S S M G A G G G G V P E N I G V K
361	AAGGCTCTTGTCTTCTACACAGGCAAGCCTCCTAAGGGAGTCAAAACCAGTTGGATCATG
121	K A L V F Y T G K P P K G V K T S W I M
421	CATGAATATCGCCTCACTGAGACCCCCAACAACTTCATTGTCAACAAGCCAATGAAGCCT
141	H E Y R L T E T P N N F I V N K P M K P
481	AAAGACTCATCCATGAGGTTGGATGATTGGGTTCTATGTCGAATCTACCGCAAACACAAT
161	K D S S M R L D D W V L C R I Y R K H N
541	TCTTCTCCAAGTTCAGCAGCAGCAACAAGTGATCAAGACCAAGAAGAAGAAGATGAACAA
181	S S P S S A A A T S D Q D Q E E E D E Q
601	TTTGTCCAAGAAACCCTTTTACCAATCCTGAAAAGTCCTCCAAGGAACGCCACTCTCAAT
201	F V Q E T L L P I L K S P P R N A T L N
661	CCTCAAAAGTCCTCTTCCTTTTCCAACTTACTGGATGCCATGGACTACTCAATGTTGAGT
221	P Q K S S S F S N L L D A M D Y S M L S
721	AGCTTCCTGTCTGACAATCAAATGTACCAAACCGGGTTTGAGCCAATTCCTATGCACACT
241	S F L S D N Q M Y Q T G F E P I P M H T
781	TCTGGACTCCTAGACCAGCCTTGCTTCAACAATGGCACCAACAATGGTTGCAGTAGCAGC
261	S G L L D Q P C F N N G T N N G C S S S
841	TTTATGCTTCAAAAGCTACCTCAGTTGAACTCTCCTCTTCTGGACAACGTGGAGAACAAA
281	F M L Q K L P Q L N S P L L D N V E N K
901	CTCAAGCGCCCACATTCAAGTGTAGATGAGCTCATGTTCCACCCATCAAAGAAACAACAC
301	L K R P H S S V D E L M F H P S K K Q H
961	ATCAGCTATTCTTGCACTACTTTGACTAACACAGCGAGTACCCATGATCATCAATCTGAC
321	I S Y S C T T L T N T A S T H D H Q S D
1021	CCTCTTCAGTACAACTTTCTACACCAGTCATTGTTGAATCCGCAGTTACTTTTGAGCCCT
341	P L Q Y N F L H Q S L L N P Q L L L S P
1081	CATCTCCAGTTCCAAGGATAA
361	H L Q F Q G *
	GTGATCAACCAATCTAAAGAAGTCTAAAACGGGAAATTTGAAAAAAAAAAAAAAAAACGAAA
	ACAAAAACGAAAACCGTAAAAAGTATTGGAAAAAAAAAAAACGCAAAACTTATTGAGGTAGC
	CAAGGCCCTGATGAAGATGTGTACAAATTATTTTGTGTGATTTTTTGTGTTGGAAAATGAC
	ACGGTTGAATTCTAGTCATATTGAACTACTTTAACCATGGAATCAAAAATATGATACCATG
	TTAAAGTCAAAAGTTGGTGGAAAAAACAAAATTGCACCAAGGGAGTTGATAAAGTAAAATC
	ATATATATATATATATATATATA
	3’ UTR
