# Supplementary material for: LcNAC13 Is Involved in the Reactive Oxygen Species-Dependent Senescence of the Rudimentary Leaves in Litchi chinensis
Source: Front Plant Sci. 2022 May 9;13:886131. doi: 10.3389/fpls.2022.886131 (PMC9125249; doi:10.3389/fpls.2022.886131)
Supplement: Supplementary file 8 [file Data_Sheet_4.PDF]

Table S3 Enriched biological process potentially involved in *LcNAC13*-silenced rudimentary leaves in litchi

| | Gene Ontology term | Cluster frequency | Genome frequency of use | P-value | Corrected P-value |
|---|---|---|---|---|---|
| GO:0034645 | cellular macromolecule biosynthetic process | 9 of 126 in the list | 1034 of 12082 in the genome | 0.76127 | 1 |
| GO:0009908 | flower development | 2 of 126 in the list | 96 of 12082 in the genome | 0.26465 | 1 |
| GO:0010468 | regulation of gene expression | 8 of 126 in the list | 1194 of 12082 in the genome | 0.93919 | 1 |
| GO:0032502 | developmental process | 8 of 126 in the list | 1274 of 12082 in the genome | 0.96233 | 1 |
| GO:0044699 | single-organism process | 78 of 126 in the list | 6712 of 12082 in the genome | 0.08761 | 1 |
| GO:0000160 | phosphorelay signal transduction system | 3 of 126 in the list | 51 of 12082 in the genome | 0.01602 | 1 |
| GO:0010467 | gene expression | 3 of 126 in the list | 989 of 12082 in the genome | 0.99847 | 1 |
| GO:0071554 | cell wall organization or biogenesis | 7 of 126 in the list | 251 of 12082 in the genome | 0.01604 | 1 |
| GO:0003006 | developmental process involved in reproduction | 7 of 126 in the list | 567 of 12082 in the genome | 0.37925 | 1 |
| GO:0005975 | carbohydrate metabolic process | 7 of 126 in the list | 791 of 12082 in the genome | 0.72616 | 1 |
| GO:0051707 | response to other organism | 6 of 126 in the list | 364 of 12082 in the genome | 0.18015 | 1 |
| GO:0009628 | response to abiotic stimulus | 6 of 126 in the list | 844 of 12082 in the genome | 0.88198 | 1 |
| GO:0009267 | cellular response to starvation | 4 of 126 in the list | 68 of 12082 in the genome | 0.00549 | 1 |
| GO:0042594 | response to starvation | 4 of 126 in the list | 68 of 12082 in the genome | 0.00549 | 1 |
| GO:0031667 | response to nutrient levels | 4 of 126 in the list | 71 of 12082 in the genome | 0.00639 | 1 |
| GO:0042743 | hydrogen peroxide metabolic process | 4 of 126 in the list | 144 of 12082 in the genome | 0.06374 | 1 |
| GO:0072593 | reactive oxygen species metabolic process | 4 of 126 in the list | 166 of 12082 in the genome | 0.09553 | 1 |
| GO:1901700 | response to oxygen-containing compound | 4 of 126 in the list | 426 of 12082 in the genome | 0.65367 | 1 |
| GO:0009416 | response to light stimulus | 4 of 126 in the list | 443 of 12082 in the genome | 0.68379 | 1 |
| GO:0009692 | ethylene metabolic process | 3 of 126 in the list | 27 of 12082 in the genome | 0.00269 | 0.68797 |
| GO:0006979 | response to oxidative stress | 3 of 126 in the list | 155 of 12082 in the genome | 0.2197 | 1 |
| GO:0048513 | organ development | 3 of 126 in the list | 387 of 12082 in the genome | 0.77338 | 1 |

| | | | | | |
|---|---|---|---|---|---|
| GO:0048731 | system development | 3 of 126 in the list | 480 of 12082 in the genome | 0.88218 | 1 |
| GO:0048437 | floral organ development | 2 of 126 in the list | 84 of 12082 in the genome | 0.21833 | 1 |
| GO:0090567 | reproductive shoot system development | 2 of 126 in the list | 129 of 12082 in the genome | 0.3904 | 1 |
| GO:0010941 | regulation of cell death | 2 of 126 in the list | 130 of 12082 in the genome | 0.39409 | 1 |
| GO:0043067 | regulation of programmed cell death | 2 of 126 in the list | 130 of 12082 in the genome | 0.39409 | 1 |
| GO:0009755 | hormone-mediated signaling pathway | 2 of 126 in the list | 282 of 12082 in the genome | 0.79696 | 1 |
| GO:0032870 | cellular response to hormone stimulus | 2 of 126 in the list | 282 of 12082 in the genome | 0.79696 | 1 |
| GO:0009725 | response to hormone | 2 of 126 in the list | 389 of 12082 in the genome | 0.91705 | 1 |
