# Supplementary material for: LcNAC13 Is Involved in the Reactive Oxygen Species-Dependent Senescence of the Rudimentary Leaves in Litchi chinensis
Source: Front Plant Sci. 2022 May 9;13:886131. doi: 10.3389/fpls.2022.886131 (PMC9125249; doi:10.3389/fpls.2022.886131)
Supplement: Supplementary file 9 [file Data_Sheet_5.PDF]

Table S4 Total effects and Bootstrapping analysis in PLS-SEM.

Pathways during ABA modle	Original Sample (Total effects)	Sample Mean	Standard Deviation	Standard Error	T Statistics
**ABA -> MYB**	0.992137	0.992124	0.000471	0.000471	2107.529195
**ABA -> NAC**	0.983165	0.983157	0.001018	0.001018	966.216072
**ABA -> WRKY**	0.983229	0.983185	0.001646	0.001646	597.522439
**ABA -> senescence**	0.969450	0.969511	0.002247	0.002247	431.451733
**NAC -> senescence**	0.268635	0.265770	0.062760	0.062760	4.280368
**WRKY -> senescence**	0.932958	0.936473	0.101459	0.101459	9.195389

Pathways during Auxin modle	Original Sample (Total effects)	Sample Mean	Standard Deviation	Standard Error	T Statistics
**Auxin -> MYB**	0.991181	0.991191	0.000536	0.000536	1847.639264
**Auxin -> NAC**	0.991200	0.991158	0.000936	0.000936	1059.193023
**Auxin -> WRKY**	0.981084	0.981080	0.001374	0.001374	714.242672
**Auxin -> senescence**	0.976278	0.976316	0.001498	0.001498	651.928197
**MYB -> senescence**	0.691522	0.696664	0.121644	0.121644	5.684790
**NAC -> senescence**	0.923175	0.934280	0.210244	0.210244	4.390977
**WRKY -> senescence**	-0.022758	-0.035227	0.204641	0.204641	0.111211

Pathways during CTK modle	Original Sample (Total effects)	Sample Mean	Standard Deviation	Standard Error	T Statistics
**CTK -> MYB**	-0.909092	-0.909259	0.011798	0.011798	77.057966
**CTK -> NAC**	-0.906257	-0.906615	0.009210	0.009210	98.396262
**CTK -> WRKY**	-0.922523	-0.922766	0.008037	0.008037	114.781785
**CTK -> senescence**	-0.915273	-0.915384	0.010159	0.010159	90.095454
**MYB -> senescence**	0.345117	0.348104	0.062354	0.062354	5.534778
**NAC -> senescence**	-0.178488	-0.182105	0.116768	0.116768	1.528566
**WRKY -> senescence**	0.830952	0.832024	0.122277	0.122277	6.795650

Pathways during GA modle	Original Sample (Total effects)	Sample Mean	Standard Deviation	Standard Error	T Statistics
**GA -> MYB**	0.987440	0.987439	0.001372	0.001372	719.567064
**GA -> NAC**	0.984297	0.984379	0.000669	0.000669	1471.694213
**GA -> WRKY**	0.992255	0.992283	0.000427	0.000427	2323.635250
**GA -> senescence**	0.990469	0.990470	0.000816	0.000816	1213.633409
**MYB -> senescence**	0.023109	0.022631	0.092462	0.092462	0.249926

**NAC -> senescence**	0.605641	0.608161	0.133971	0.133971	4.520691
**WRKY -> senescence**	-0.200535	-0.204434	0.181883	0.181883	1.102546

Pathways during JA modle	Original Sample (Total effects)	Sample Mean	Standard Deviation	Standard Error	T Statistics
**JA -> MYB**	0.985364	0.985401	0.000968	0.000968	1017.998222
**JA -> NAC**	0.981769	0.981725	0.001856	0.001856	528.988319
**JA -> WRKY**	0.970425	0.970450	0.002306	0.002306	420.885036
**JA -> senescence**	0.972447	0.972507	0.001473	0.001473	660.178294
**MYB -> senescence**	0.513868	0.514560	0.071340	0.071340	7.203049
**NAC -> senescence**	0.646048	0.645110	0.066312	0.066312	9.742518

T statistics higher than 1.96 are significant at 5% (Hair et al., 2011).
