# Supplementary material for: LcNAC13 Is Involved in the Reactive Oxygen Species-Dependent Senescence of the Rudimentary Leaves in Litchi chinensis
Source: Front Plant Sci. 2022 May 9;13:886131. doi: 10.3389/fpls.2022.886131 (PMC9125249; doi:10.3389/fpls.2022.886131)
Supplement: Supplementary file 10 [file Data_Sheet_6.PDF]

Table S5 PLS-SEM results quality criteria.

quality criteria of ABA modle	AVE	Composite Reliability	R Square
**ABA**	0.832893	0.936815	
**MYB**	0.741413	0.910728	0.984337
**NAC**	0.806001	0.971695	0.966614
**WRKY**	0.827956	0.971672	0.966740
**senescence**	0.793376	0.307263	0.986457

quality criteria of Auxin modle	AVE	Composite Reliability	R Square
**Auxin**	0.887409	0.996211	
**MYB**	0.741434	0.910959	0.982441
**NAC**	0.806018	0.971821	0.982476
**WRKY**	0.827903	0.971740	0.962526
**senescence**	0.793255	0.317446	0.988412

quality criteria of CTK modle	AVE	Composite Reliability	R Square
**CTK**	0.907137	0.966983	
**MYB**	0.741106	0.907695	0.826448
**NAC**	0.805876	0.971420	0.821301
**WRKY**	0.827862	0.971390	0.851048
**senescence**	0.793527	0.273236	0.985824

quality criteria of GA modle	AVE	Composite Reliability	R Square
**GA**	0.801199	0.725430	
**MYB**	0.741398	0.909311	0.975038
**NAC**	0.805958	0.971567	0.968841
**WRKY**	0.827948	0.971550	0.984571
**senescence**	0.793507	0.290423	0.988604

quality criteria of JA modle	AVE	Composite Reliability	R Square
**JA**	0.909347	0.983650	
**MYB**	0.741405	0.911218	0.970941
**NAC**	0.805989	0.971883	0.963870
**WRKY**	0.827634	0.971869	0.941725
**senescence**	0.793026	0.330750	0.985458

All latent variables are significant and goodness-of-fit measures when Average variance extracted

(AVE; Indicator for converge validity) and composite reliability (indicator for internal consistency reliability) are equal or higher than 0.5 and 0.7 (Hair et al., 2011).
