# Supplementary material for: LcNAC13 Is Involved in the Reactive Oxygen Species-Dependent Senescence of the Rudimentary Leaves in Litchi chinensis
Source: Front Plant Sci. 2022 May 9;13:886131. doi: 10.3389/fpls.2022.886131 (PMC9125249; doi:10.3389/fpls.2022.886131)
Supplement: Supplementary Table 6 — Genes information in Figure 10. [file Data_Sheet_3.PDF]

Number	Genes name	Forward primer（5'-3'）	Reverse primer（5'-3'）	
1	LcActin-F/R	ACCGTATGAGCAAGGAAATCACTG	TCGTCGTACTCACCCTTTGAAATC	
2	LcNAC13-F/R	ATGGTTCACACGAAGAACCCTG	TTATCCTTGGAACTGGAGATGAG	
3	pBI121-LcNAC13-F/R	GAACACGGGGGACTCTAGAATGGTTCACACGAAGAACCCTG	GGCGTCTTCCATGGATCCTCCTTGGAACTGGAGATGAG	
4	35S:LcNAC13-F/R	GCACAATCCCACTATCCTTCG	TTATCCTTGGAACTGGAGATGAG	
5	NptIIu-F/R	GTGGAGAGGCTATTCGGCTATGACTG	AGCTCTTCAGCAATATCACGGGTAGC	
6	pBI121:LcNAC13:LUC-F/R	GAACACGGGGGACTCTAGAATGGTTCACACGAAGAACCCTG	GAACACGGGGGACTCTAGAATGGTTCACACGAAGAACCCTG	
7	pTRV2-LcNAC13 F/R	TACCGAATTCTCTAGACCTCTTCCTTTTCCAACTTACTG	CTTCGGGACATGCCCGGGTCGCTGTGTTAGTCAAAGTAGTG	
